# Supplementary material for: Chaperone-mediated autophagy in fish: A key function amid a changing environment
Source: Autophagy Rep. 2024 Nov 1;3(1):2403956. doi: 10.1080/27694127.2024.2403956 (PMC11864646; doi:10.1080/27694127.2024.2403956)
Supplement: Schnebert_SupFig_R.pdf [file KAUO_A_2403956_SM8798.pdf]

FigS1

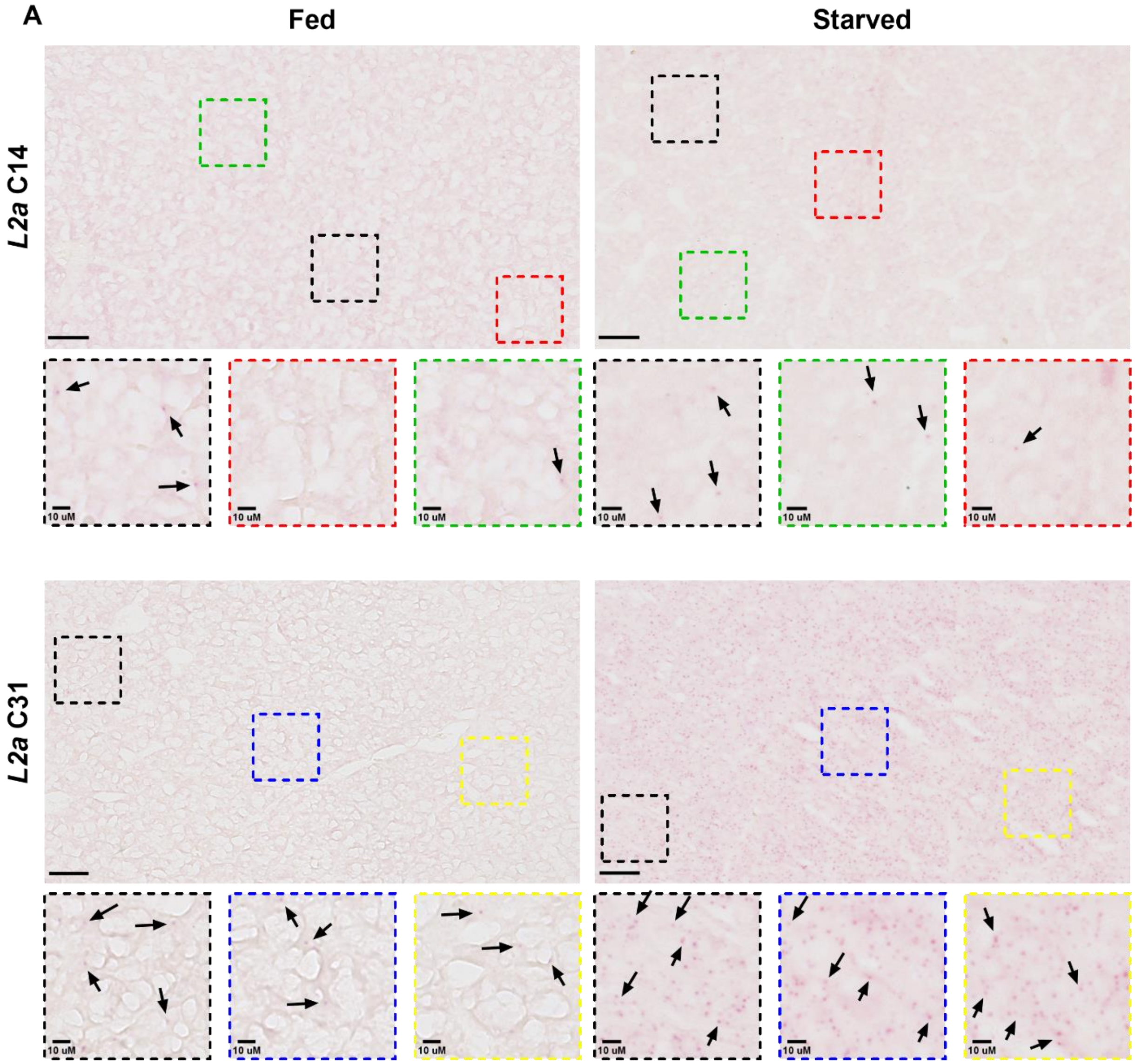

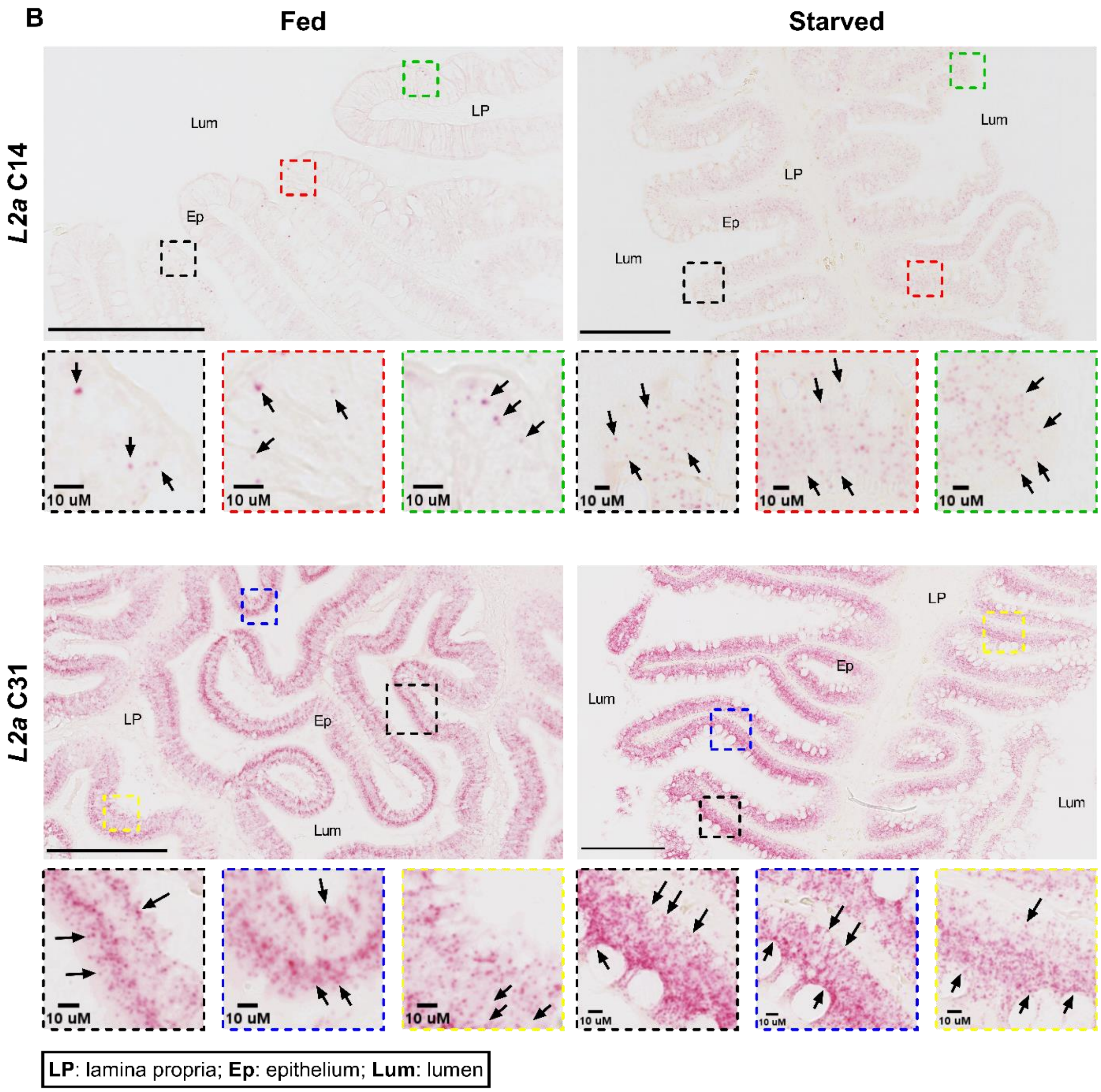
B
Fed
Starved
L2a C14
L2a C31
Lum
LP
Ep
10 uM
LP: lamina propria; Ep: epithelium; Lum: lumen

FigS2

A

```
        WT    ...TTAGCTGCAATAAATGGGAATGGAAATTGTGCATAAACTGGCGTGGACCATTTGTAATATTTTACAATATTATTGGTCCCCCGGACATTTTAGTAAA
L2AC31-KO    ...TTAGCTGCAATAAATGGGAATGGAAATTGTGCATAAACTGGCGTGGACCATTTGTAATATTTTACAATATTATTGGTCCCCCGGACATTTTAGTAAA
                ***************************************************************************************************

        WT    TCCCTCCTTTGTTTTCCAACTATTCTTGACTGAGAAGTGTCTATGATTGACCATGCATTCACCATTTTGATGTTTTACCTTTCTTCCACCCTGCAACCCC
L2AC31-KO    TCCCTCCTTTGTTTTCCAACTATTCTTGACTGAGAAGTGTCTATGATTGACCATGCATTCACCATTTTGATGTTTTACCTTTCTTCCACCCTGCAACCCC
             ****************************************************************************************************

        WT    CCCGTGCCCACTTTCTTTACTGTCATCCAGCGGAGGATTGCCAAGACGATACGACAGAGAGCTGGCTTGTTCCTATAGCGGTCGGGGTTGCCTTGACTTT
L2AC31-KO    CCCGTG----------------------------------------------------------------------------------------------
             ******

        WT    ACTGGTCCTCATTGTGTTGGTTGCCTATTTCATTGGAAGAAAGCGAAACCAGGGCACTGGCTATGAGCACTTCTAAATTATCTTCACTATGCTGAGGCTA
L2AC31-KO    ----------------------------------------------------------------------------------------------------

        WT    TAACTTCGGTCATCTGGATAATTGTTGATTTAATTTGACTAATATACTGTGCAGAGTTTACTTAATGGTGAAATTCACCGGATGGAAAGCTCTTTCTCAT
L2AC31-KO    ----------------------------------------------------------------------------------------------------

        WT    GAAACAATGACTTGGAGAAGCGGCTGTGTTAACGATGGCATGTAACAGCTGTGTGTGTAATTAGAATTTATTGATTTTGATTGACACATCTTATATTCCT
L2AC31-KO    ----------------------------TTAACGATGGCATGTAACAGCTGTGTGTGTAATTAGAATTTATTGATTTTGATTGACACATCTTATATTCCT
                                         ************************************************************************

        WT    CATCACATGGTTGACTATAGATGGCTGATGACCAAATGTTTTCCTAATTGCAATTAATGCCCATGCTCTGACGGACCCTGATGAATATGTCTTGGTT...
L2AC31-KO    CATCACATGGTTGACTATAGATGGCTGATGACCAAATGTTTTCCTAATTGCAATTAATGCCAATGCTCTGACGGACCCTGATGAATATGTCTTGGTT...
             *************************************************************************************************
```

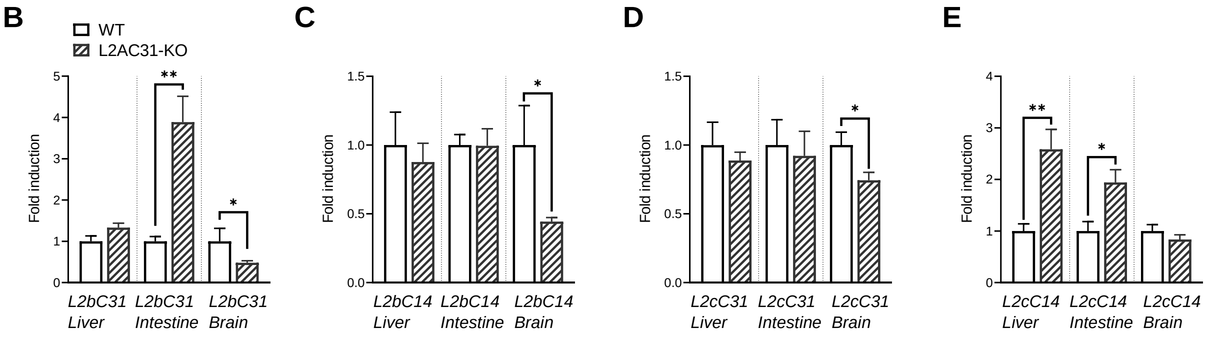

Fig S3

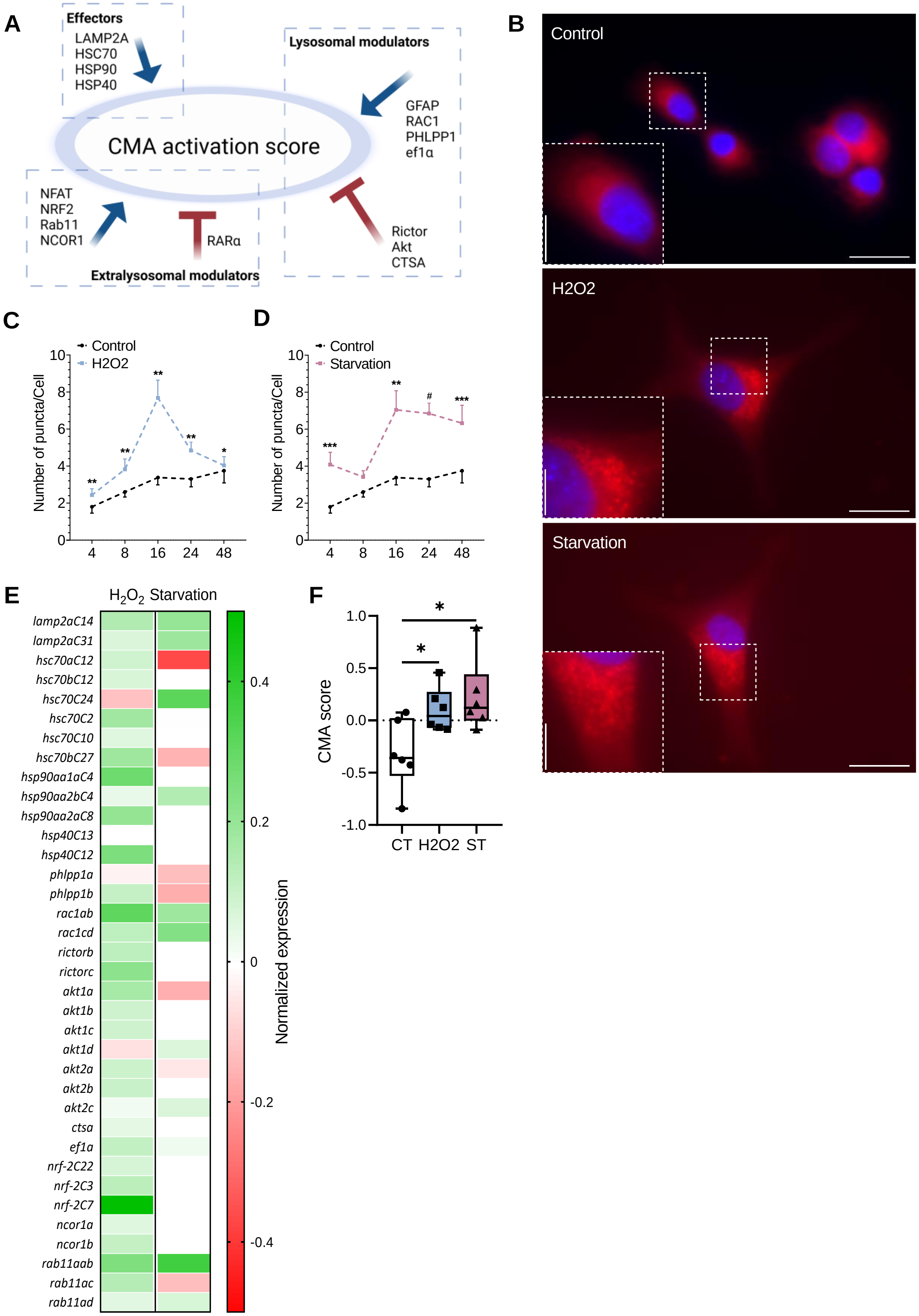
A
Effectors
LAMP2A
HSC70
HSP90
HSP40
Lysosomal modulators
GFAP
RAC1
PHLPP1
ef1α
CMA activation score
NFAT
NRF2
Rab11
NCOR1
RARα
Rictor
Akt
CTSA
Extralysosomal modulators
B
Control
H2O2
Starvation
C
Control
H2O2
Number of puncta/Cell
D
Control
Starvation
Number of puncta/Cell
E
H2O2 Starvation
lamp2aC14
lamp2aC31
hsc70aC12
hsc70bC12
hsc70C24
hsc70C2
hsc70C10
hsc70bC27
hsp90aa1aC4
hsp90aa2bC4
hsp90aa2aC8
hsp40C13
hsp40C12
phlpp1a
phlpp1b
rac1ab
rac1cd
rictorb
rictorc
akt1a
akt1b
akt1c
akt1d
akt2a
akt2b
akt2c
ctsa
ef1a
nrf-2C22
nrf-2C3
nrf-2C7
ncor1a
ncor1b
rab11aab
rab11ac
rab11ad
Normalized expression
F
CMA score
CT H2O2 ST

FigS4

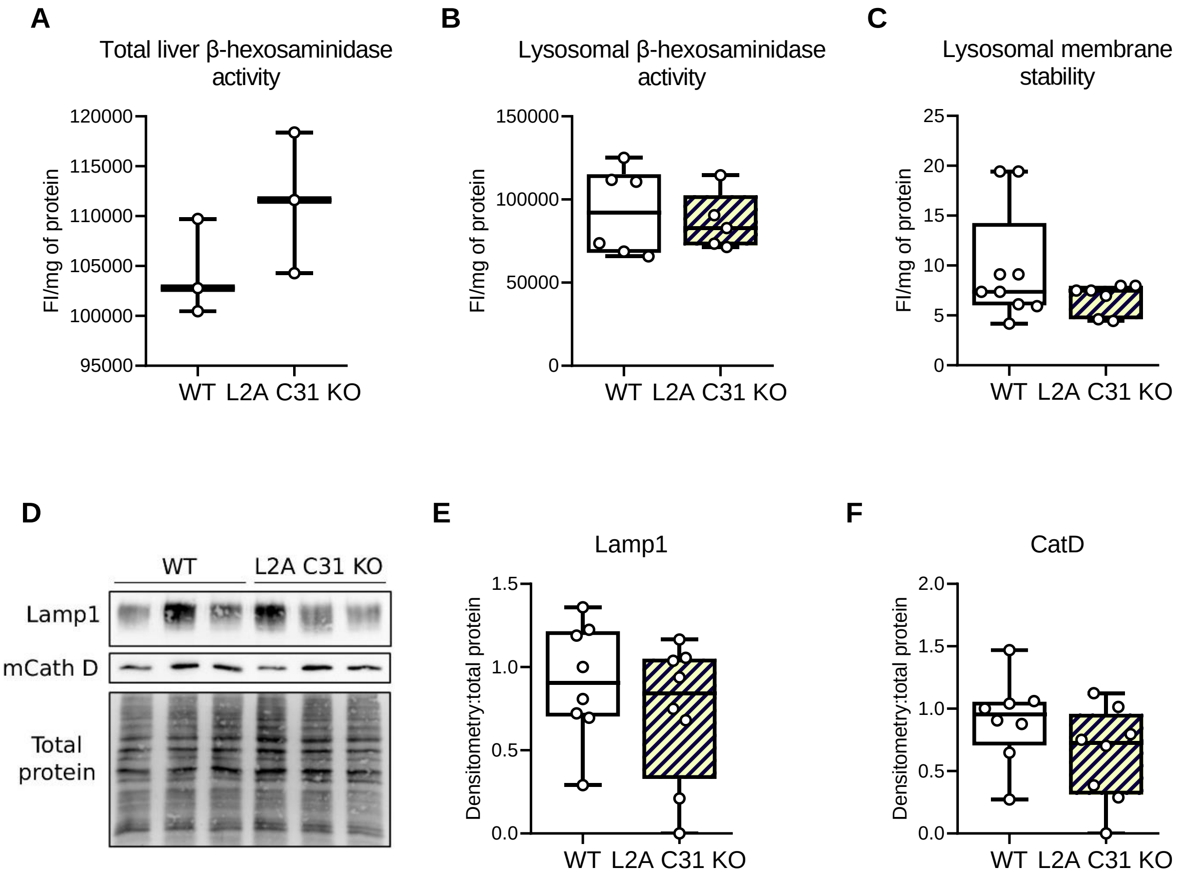
A
Total liver β-hexosaminidase activity
FI/mg of protein
120000
115000
110000
105000
100000
95000
WT
L2A C31 KO
B
Lysosomal β-hexosaminidase activity
FI/mg of protein
150000
100000
50000
0
WT
L2A C31 KO
C
Lysosomal membrane stability
FI/mg of protein
25
20
15
10
5
0
WT
L2A C31 KO
D
WT
L2A C31 KO
Lamp1
mCath D
Total protein
E
Lamp1
Densitometry:total protein
1.5
1.0
0.5
0.0
WT
L2A C31 KO
F
CatD
Densitometry:total protein
2.0
1.5
1.0
0.5
0.0
WT
L2A C31 KO

FigS5

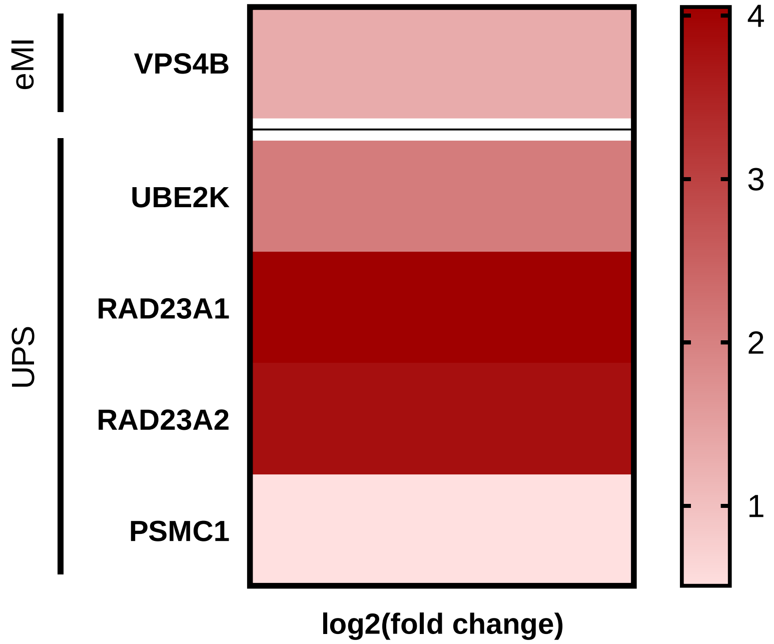
eMI
UPS
VPS4B
UBE2K
RAD23A1
RAD23A2
PSMC1
4
3
2
1
log2(fold change)
